# Hearing Characteristics of Adults before Exposure to Potentially Ototoxic Chemotherapy

**DOI:** 10.1055/s-0046-1819640

**Published:** 2026-04-30

**Authors:** Cecília Vieira Peruch, Vera Beatris Martins, Felipe de Oliveira Goulart, Marcia Salgado Machado, Eliane Dallegrave, Monalise Costa Batista Berbert

**Affiliations:** 1 Speech Therapy Department, Universidade Federal de Ciências da Saúde de Porto Alegre (UFCSPA), Porto Alegre, RS, Brazil; 2 Speech Therapy Department, Santa Casa de Porto Alegre, Porto Alegre, RS, Brazil; 3 Department of Pharmacosciences, Universidade Federal de Ciências da Saúde de Porto Alegre (UFCSPA), Porto Alegre, RS, Brazil

**Keywords:** audiology, chemotherapy, ototoxicity, oncology

## Abstract

**Introduction:**

Cancer patients undergoing potentially-ototoxic chemotherapy may present a series of hearing disorders and risk factors prior to treatment. Thus, it is necessary to know the profile of these patients to promote preventive actions and guidelines regarding the hearing health of these individuals.

**Objective:**

To describe and relate the hearing characteristics of patients entering a chemotherapy service before the exposure to potentially-ototoxic drugs.

**Methods:**

We conducted a cross-sectional study of 261 individuals who started chemotherapy between April and December 2022. All patients filled out a hearing case history and underwent otoscopy. Individuals who had a non-altered otoscopy due to obstructive cerumen underwent distortion-product otoacoustic emissions (DPOAEs) at 2, 4, 6, 8, 10, and 12 kHz in both ears.

**Results:**

In total, 83% of the patients reported good hearing and 31% described some level of hearing impairment. The most prevalent self-reported hearing issue was tinnitus (35%). Only 12% had a medical diagnosis of hearing loss, and 20% had obstructive cerumen during otoscopy. Individuals who reported hearing impairments such as reduced hearing and difficulty understanding noises demonstrated fewer responses in the DPOAEs at various frequencies. In addition, there were significant differences in DPOAE amplitudes among different age groups. Given the substantial burden of hearing loss with ageing, attention to this population is essential for the possibility of preventing or delaying hearing loss.

**Conclusion:**

Cancer patients entering a chemotherapy service have a hearing case history of risk factors and hearing alterations prior to exposure to potentially-ototoxic drugs.

## Introduction

Ototoxicity is defined as the tendency of certain drugs and other chemical substances to cause functional impairment and cellular degeneration of the tissues of the inner ear. In most severe cases, the ototoxicity mechanisms can lead to significant reduction in hearing capacity or even complete deafness. 1

For cancer patients with life-threatening illnesses who take ototoxic drugs, communication skills are central to quality of life. All hearing monitoring requires an initial assessment. Established tests should be carried out prior to any administration of potentially-ototoxic drugs so that later assessments can have a comparison basis. 2 Given the high incidence of preexisting hearing loss in the general population, the lack of initial pretreatment assessments makes it substantially harder to establish a clear association between treatments and drug-induced hearing loss. 3 4

In terms of hearing monitoring, distortion-product otoacoustic emissions (DPOAEs) are the most suitable test due to their objectivity, and they can be performed within minutes in a hospital setting. 5 Monitoring symptoms is also essential, as any alterations should be considered.

Initial audiological monitoring is essential to provide reliable basal references. Determining hearing changes even before exposure to drugs contributes significantly to a better understanding of the associated risk characteristics, identifying how hearing loss manifests itself in this population. However, while protocols emphasize posttreatment monitoring, there is a paucity of data systematically characterizing the preexisting hearing profile—including subjective complaints and objective measures such as DPOAEs—of patients at the point of entry into chemotherapy services. Considering these circumstances, the present study aimed to analyze the profile and hearing characteristics of individuals before undergoing potentially-ototoxic chemotherapy treatment.

## Methods

The current is a cross-sectional study approved by the institutional Ethics in Research Committee (under opinion no. 5.117.189), and it was registered in the National System for the Registration of Research with Human Beings in Brazil (Plataforma Brasil; CAAE:52572021.2.0000.5335). The study population was composed of individuals entering chemotherapy treatment. The sample consisted of adult patients in the chemotherapy service who started chemotherapy at a reference oncology hospital in southern Brazil between April and December 2022. A sample size of 122 subjects was calculated to test whether there is an association between EOAPD results and 4 auditory complaints (with a 10% increase due to possible losses and refusals, this number should be 136). The calculation considered a power of 80%, a significance level of 5%, and a Cohen's w effect size of 0.3 and 3 degrees of freedom at the researcher's discretion, due to the lack of similar studies that could be used as guides. This calculation was performed using the online version of the Power and Sample Size for Health Researchers (PSS Health) tool. 6 The inclusion criteria were age ≥ 18 years and no history of chemotherapy. All individuals read and received explanations regarding the research procedures and gave their participation consent by signing the Free and Informed Consent Form. Patients with medical history of neoplasms of the ear or adjacent structures were excluded from the study.

Initially, a questionnaire was developed by the authors to verify hearing symptoms or complaints. Information such as type of neoplasm, location, staging, age, gender, ethnicity, comorbidities, and associated pathologies were obtained from medical records.

Visual inspection of the external acoustic meatus was conducted with an otoscope (Omni 3000 Xenon, Medical Devices [Pvt] Ltd.) to examine any occurrences that might prevent the hearing tests from being performed. If obstructive cerumen was found, the patient was advised to consult an otorhinolaryngologist. In such cases, the patients answered the questionnaire but did not undergo DPOAEs. Individuals with normal meatoscopy underwent DPOAEs using the Interacoustics A/S Otoread Clinical device, which is designed to capture responses through a miniaturized microphone housed in the external acoustic meatus sealed by a latex ear cup. The DPOAEs were recorded at 2, 4, 6, 8, 10 and 12 kHz in both ears. The recordings were conducted with the patients sitting on a stretcher or armchair in the chemotherapy service room. F1 and F2 tones were presented at a stimulus level of 65 and 55dB SPL. Signal-to-noise ratios (S/N) were documented in their entirety to conduct the analysis. The results were printed directly on the equipment, scanned and stored in hard copy alongside the informed consent form. The researcher responsible for the tests underwent 4 hours of formal training beforehand and had more than 7 years of experience in audiology.

The data obtained was reviewed by the researcher in charge prior to its transfer to a Google (Alphabet Inc.) spreadsheet. Individual S/N results were registered by frequency range and paired with data from each patient's hearing case history. The DPOAE S/N values were analyzed in two ways: firstly, by assessing the presence or absence of responses. The criterion to classify responses as *present* was a minimum of 6 dB or higher above the noise indicator. Secondly, the numerical value of the S/N was used to cross-reference age groups.

For the statistical analysis, the categorical variables were organized by absolute frequency and proportion, and statistical comparisons were made using the Chi-squared test of independence or the Fisher's exact test. The quantitative variable used was the DPOAE response frequency, expressed as by mean and standard deviation values and compared among age groups using the Kruskal-Wallis non-parametric test, followed by post-hoc analysis using the Mann-Whitney test with Bonferroni correction. The non-parametric technique was chosen due to the non-normal distribution of the variable and absolute frequencies below 30 dB in some age groups. All statistical analyses were performed in the R (R Foundation for Statistical Computing) software, version 4.2.0, at a 95% confidence level.

## Results

The final sample included 261 patients, and their sociodemographic and oncological characteristics are shown in Table 1 . The oncological characteristics were organized into categories according to the primary location of the malignant neoplasms. The highest incidence reported was of breast cancer, followed by lung cancer.

**Table 1:** Sociodemographic and oncological characteristics

Characteristics: n (%)	n = 261
**Sex**	
Female	149 (57)
Male	112 (43)
**Age group (years)**	
18–49	65 (25)
50–59	59 (23)
60–69	86 (33)
≥ 70	51 (20)
**Color/Ethnicity**	
Yellow	3 (1.1)
White	197 (75)
Indigenous	2 (0.8)
Black	20 (7.7)
Brown	39 (15)
**Oncological topography**	
Head and neck	12 (4.6)
Colorectal	36 (14)
Upper gastrointestinal	31 (12)
Gynecological	21 (8.0)
Hepatic	3 (1.1)
Lymphoma	22 (8.4)
Breast	62 (24)
Melanoma	6 (2.3)
Lung	49 (19)
Sarcoma	4 (1.5)
Urological	15 (5.7)

The self-reported hearing symptoms are presented in Table 2 ; the data are divided by individuals with normal and altered meatoscospy to exclude any bias risk and enable a more in-depth analysis of their complains. In the group with no alterations, 172 (82%) patients considered that they heard well, while 59 (28%) felt their hearing had decreased over time, and 155 (74%) reported speech-in-noise (SiN) impairment; correlations of these findings will be explored throughout the results. Tinnitus was the most common self-reported symptom, experienced by 78 (39%) individuals. Only 31 (12%) patients reported having been diagnosed with hearing loss by a medical professional, and 2 (0.8%) were hearing-aid users. Among the 261 patients, 51 (20%) had altered meatoscopy due to obstructive cerumen—of these, 31 (12%) individuals presented obstruction in both ears, 13 (5.0%), in the right ear, and 7 (2.7%), in the left ear. The data are not too different in this group: 44 (86%) patients considered that they heard well, 21 (41%) felt their hearing had decreased over time, 30 (59%) reported SiN impairment, and 14 (27%) had experienced tinnitus.

**Table 2:** Self-reported hearing symptoms

	Meatoscopy: n (%)	
Questionnaire	Normal (n = 210)	Altered (n = 51)
Considered that they hear well	172 (82)	44 (86)
Felt that hearing had decreased over time	59 (28)	21 (41)
Speech-in-noise impairment	155 (74)	30 (59)
Tinnitus	78 (37)	14 (27)
Hearing loss diagnosis	29 (14)	2 (3,9)
Hearing-aid user	2 (1,0)	0 (0)
Family history of hearing loss < 50 years	23 (11)	10 (20)
Occupational noise exposure	107 (51)	29 (57)
Otitis history	35 (17)	7 (14)
Otologic surgery	4 (1,9)	0 (0)

Patients with altered meatoscopy due to obstructive cerumen only answered the questionnaire and exhibited a statistically significant difference ( *p* < 0.05) in terms of the difficulty in understanding SiN.

Table 3 compares the DPOAE responses by presence and absence of self-reported complaints, with each line representing the proportion of otoacoustic emissions (≥ 6) within each category (yes/no) of the 4 self-reported complaint questions divided by ear, referred as right ear (RE) and left ear (LE). When asked if they had good hearing, patients who answered negatively had fewer DPOAEs found at the frequencies of 4, 6, and 8kHz in the LE and at 2, 4, 6, 8, and 10 kHz in the RE when compared to patients who answered positively ( *p* < 0.05). Similarly, patients who considered their hearing to be reduced had fewer DPOAEs at 2, 4, 6, 8, and 10 kHz in the RE, and at 2, 4, 6, and 8 kHz in the LE.

**Table 3:** Comparison of DPOAE responses by presence and absence of self-reported complaints

DPOAEs (≥ 6)	Total: n (%) (n 210)	Considered that they hear well: n (%)			Felt that hearing had decreased over time: n (%)			Speech-in-noise impairment: n (%)			Tinnitus: n (%)		
		No (n = 38)	Yes (n = 172)	*p* -value	No (n = 151)	Yes (n = 59)	*p* -value	No (n = 55)	Yes (n = 155)	*p* -value	No (n = 132)	Yes (n = 78)	*p* -value
**LE: 2 kHz**	139 (66)	20 (53)	119 (69)	0.051	108 (72)	31 (53)	0.009*	42 (76)	97 (63)	0.063	87 (66)	52 (67)	0.91
**LE: 4 kHz**	125 (60)	9 (24)	116 (67)	< 0.001*	110 (73)	15 (25)	< 0.001*	43 (78)	82 (53)	0.001*	86 (65)	39 (50)	0.031*
**LE: 6 kHz**	91 (43)	7 (18)	84 (49)	< 0.001*	84 (56)	7 (12)	< 0.001*	33 (60)	58 (37)	0.004*	66 (50)	25 (32)	0.011*
**LE: 8 kHz**	83 (40)	8 (21)	75 (44)	0.01*	70 (46)	13 (22)	0.001*	30 (55)	53 (34)	0.008*	61 (46)	22 (28)	0.01*
**LE: 10 kHz**	75 (36)	11 (29)	64 (37)	0.34	60 (40)	15 (25)	0.052	24 (44)	51 (33)	0.15	51 (39)	24 (31)	0.25
**LE: 12 kHz**	45 (21)	5 (13)	40 (23)	0.17	35 (23)	10 (17)	0.32	15 (27)	30 (19)	0.22	31 (23)	14 (18)	0.34
**RE: 2 kHz**	135 (64)	15 (39)	120 (70)	< 0.001*	107 (71)	28 (47)	0.001*	43 (78)	92 (59)	0.012*	84 (64)	51 (65)	0.8
**RE: 4 kHz**	146 (70)	15 (39)	131 (76)	< 0.001*	117 (77)	29 (49)	< 0.001*	48 (87)	98 (63)	<0.001*	95 (72)	51 (65)	0.32
**RE: 6 kHz**	94 (45)	8 (21)	86 (50)	0.001*	83 (55)	11 (19)	< 0.001*	34 (62)	60 (39)	0.003*	67 (51)	27 (35)	0.023*
**RE: 8 kHz**	88 (42)	10 (26)	78 (45)	0.031*	74 (49)	14 (24)	< 0.001*	30 (55)	58 (37)	0.027*	65 (49)	23 (29)	0.005*
**RE: 10 kHz**	79 (38)	8 (21)	71 (41)	0.02*	68 (45)	11 (19)	< 0.001*	24 (44)	55 (35)	0.28	56 (42)	23 (29)	0.061
**RE: 12 kHz**	45 (21)	6 (16)	39 (23)	0.35	36 (24)	9 (15)	0.17	16 (29)	29 (19)	0.11	36 (27)	9 (12)	0.007*

**Abbreviations:** DPOAE, distortion-product otoacoustic emissions; LE, left ear; RE, right ear.

**Notes:** Chi-squared test of independence; *The values in bold indicate statistical significance ( *p* -value < 0.05).

**Fig. 1:**
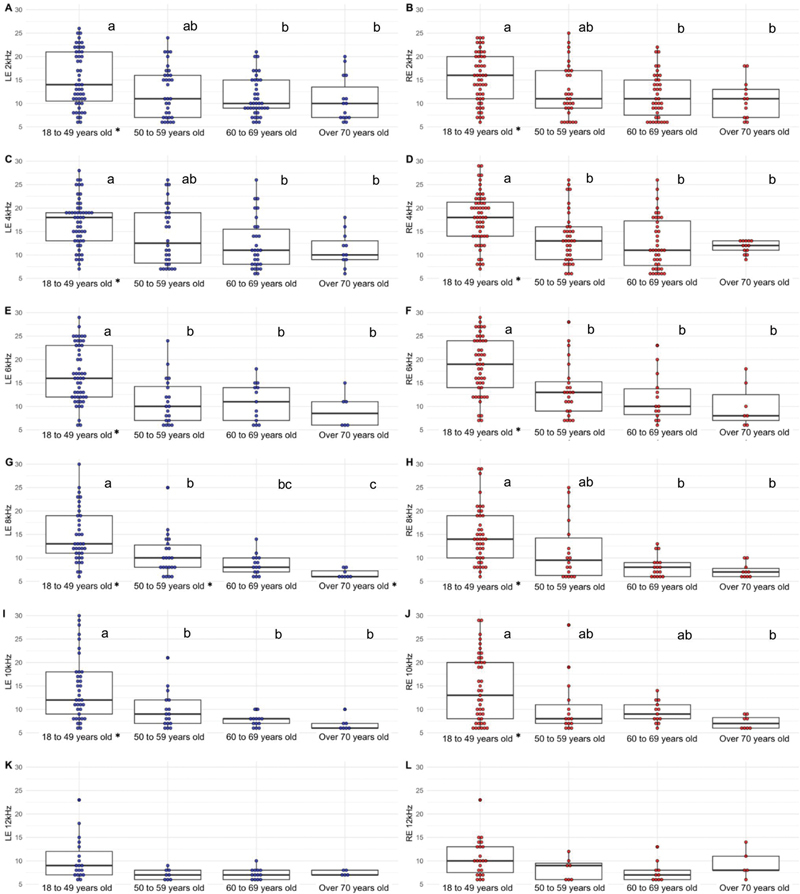
Left- and right-ear distortion-product otoacoustic emission (DPOAE) responses according to frequency and divided by age group: multiple box plot + dot plot; n = 210.

Fig. 1 compares the difference in the amplitude of DPOAE responses according to frequency ranges by age groups. The *p* -value presented in the figure refers to the result of the global Kruskal-Wallis test, in which a *p* -value < 0.05 indicates that at least one of the age groups has a DPOAE frequency value that is different from the others. The image provides a clearer visualization of the progressive decline in responses with increasing age across all tested frequencies. The older the individuals, the lower the S/N in both ears.

The post-hoc method is a complementary analysis that identifies which specific age groups differ from each other. Fig. 1 reports a lettering system that denotes these differences: if two age groups share the same superscript letter, their DPOAE frequency values are not statistically different. For example, in the first frequency shown (LE 2 kHz), the age groups 18 to 49 years and 50 to 59 years share the letter “a,” indicating that their mean frequencies were not statistically different. Similarly, the age groups 50 to 59 years, 60 to 69 years, and ≥ 70 years share the letter “b,” so they do not differ significantly from each other. However, the age groups 18 to 49 years and ≥ 70 years have no letters in common, indicating a statistically significant difference between them. Therefore, it can be concluded that the mean response frequency of LE 2 kHz is higher in the 18 to 49-year age group compared with the ≥ 70 years group. For all other frequencies in the figure, the interpretation follows the same logic. When the global *p* -value is > 0.05, no lettering system is shown, since the DPOAE frequencies did not differ statistically among the age groups.

## Discussion

The current study described the auditory anamnesis profile and DPOAE responses of cancer patients entering a chemotherapy ward. As a result, it was possible to identify risk factors in terms of auditory symptoms that can contribute to hearing loss in cancer patients exposed to ototoxic agents. Since the underlying disease is a major burden to the overall quality of life of these individuals, hearing loss may contribute negatively towards exacerbating this situation.

The results highlight the importance of a well-performed anamnesis to adequately understand the patient and the potential risk factors associated with cancer treatments. Studies 7 8 have shown that the medical history can help the patient's auditory self-perception throughout treatment, provide the healthcare professional with information about changes in responses, and enable intervention actions in the event of auditory symptoms of ototoxicity.

Breast and lung cancers were the most prevalent types, which corroborates with global statistics findings. 9 Similar data is found in Brazilian databases: Instituto Nacional do Câncer 10 (INCA) reports breast and prostate topographies as the most prevalent, respectively.

During the auditory anamnesis, exposure to noise was the predominant risk factor. Assunção et al. 11 found a 32.1% prevalence of self-reported occupational noise exposure in the Brazilian population, whereas the country's southern states have reported a prevalence higher than 37%.

The fact that 33 (13%) of the patients reported a family history of hearing loss before the age of 50 years serves as a warning sign for ototoxicity risks. Different studies 12 13 have correlated genetic factors with hearing loss and ototoxicity, which are not necessarily a determinant factor, but rather an important part of a multiple system associated with other risk factors.

The results of the current study indicated that 51 (20%) of the patients entering chemotherapy had obstructive cerumen in one or both ears. According to some authors, 14 15 this condition is more common in older adults, which corroborates the findings of the present study. Estimates suggest that between 19% and 65% of people older than 65 years have obstructive cerumen. The elderly are more likely to have drier earwax, slower skin movement, and debris outside the ear canal, which leads to an increased earwax buildup. 14 This condition may bring forth a large array of symptoms, such as tinnitus, vertigo, discharge, pain, hearing loss, blocked ears, and otitis externa. 15 Those occurrences align with the findings of the present study, in which the patients had a statistically significant SiN impairment (that is, they demonstrated difficulty in understanding speech amidst background noise). Oron et al. 15 reported that cerumen removal significantly improved the wellbeing of elderly patients, thus reinforcing the importance of ear-cleaning referrals.

Tinnitus was the most common auditory symptom reported by the patients with normal meatoscopy 78 (37%). The literature 16 17 18 shows a prevalence of 10% to 30% in adults, and as high as 60% in the elderly. Even though tinnitus is frequently related to hearing loss, individuals with normal hearing may also experience this symptom, 19 20 which corroborates the current study's data, considering 39 (50%) of the individuals had DPOAEs present at 4 kHz, 25 (32%), at 6 kHz, and 22 (28%), at 8 kHz in the LE, while 27 (35%), at 6 kHz, 23 (29%), at 8 kHz, and 9 (12%), at 12 kHz in the RE.

The study also associated the absence of DPOAE responses at different frequencies with self-reported complaints of not hearing well, loss of hearing, and SiN. The proportion of DPOAEs present in the frequencies of 4, , and 8 kHz in both ears was higher among patients who reported they heard well ( *p* < 0.001) compared to those who answered the question negatively. These findings align with those of numerous studies 21 22 that demonstrate awareness of difficulty among individuals with hearing loss.

However, 75 (44%) to 117 (77%) of the patients who reported no reduction in hearing had absent otoacoustic emissions at 4, 6, and 8 kHz. These statistical findings align with those made by Cruz et al., 23 which suggests that the prevalence of clinically-diagnosed hearing impairment may be higher when compared to studies on self-reported hearing impairment, considering that this condition's onset is often characterized as slow and gradual, especially when attributed to presbycusis. 24 25 Thus, when the elderly become aware of some level of difficulty and report hearing impairment, their condition is already far more advanced, strengthening the hypothesis that self-reported hearing loss tends to raise underestimated prevalence data. 25

As seen in previous studies, 26 27 the decrease in otoacoustic emission (OAE) responses occurs with increasing age, and the same pattern appears in the present study. Given the substantial burden of hearing loss with ageing, the possibility of preventing or delaying hearing loss is necessary. These data highlight the need for focused attention on patients older than 50 years undergoing chemotherapy, as more factors are associated with a risk to hearing health. 28 The literature 9 10 reports that the largest cancer population is older than 50 years, which corroborates the current study's findings, in which 196 (75%) of the patients were aged ≥ 50 years. These individuals are more susceptible to ototoxic drugs due to age-related impaired renal function. 28 As such, it is also important to realize that there may be confounding factors due to the presence of other auditory findings associated with the age group.

Early detection and proactive management of hearing loss is the main reason for improved monitoring, considering that hearing impairment is often overlooked by the affected individuals, which results in undertreatment outcomes, especially for patients dealing with life-threatening illnesses. 17 In fact, most individuals with hearing impairments do not seek assistance for their condition, 29 aligning with the present study's prevalence rates: 45 (18%) of the patients considered that they did not hear well, and 80 (28%) reported feeling a decrease in hearing, but only 31 (12%) were diagnosed with hearing loss, even though 94 (45%) of the patients did not present responses at the frequencies of 6 kHz or higher in the DPOAE test. Unfortunately, untreated hearing loss degrades interpersonal relationships and socioemotional well-being; 29 30 31 it hinders understanding of health and treatment-related information and is associated with increased hospital readmissions. 30 Addressing hearing loss is, therefore, especially important in critical illnesses as cancer.

Prospective monitoring of ototoxicity combined with education and counseling may help patients assess the impact of preexisting hearing loss and worsening of symptoms in their daily lives. Between 23 (61%) and 31 (82%) ( *p* < 0.05) of the patients with absent DPOAEs at 4, 6, and 8 kHz reported not hearing well. The literature 29 31 states such awareness increases the likelihood of a patient seeking hearing rehabilitation and undergoing the prescribed interventions.

The current study is part of a larger project aimed at ototoxicity identification in different chemotherapy protocols. Tests were conducted regarding the hearing characteristics of patients admitted to the chemotherapy ward to learn about the hearing characteristics of individuals undergoing potentially-ototoxic treatment. This data is significantly relevant, considering the patients included demonstrate different risk factors and signs that should be evaluated during cancer treatment. Thorough patient profiling is essential for future comparisons using preventive and corrective actions in Brazilian chemotherapy departments. Additionally, the prevalence of new patients with hearing impairments emphasizes the need for other health professional's referrals so that proper attention and possible multidisciplinary treatment for the arising conditions are correctly offered.

## Conclusion

The present study's data indicates that oncology patients being admitted into chemotherapy treatment have a hearing case history of risk factors and hearing alterations prior to exposure to potentially-ototoxic drugs that may be crucial during treatment.

Furthermore, the findings highlight the importance of advising patients, family members, and caregivers about early implementation of preventive hearing care for synergistic factors and communication strategies in order to reduce the negative effects on quality of life associated with hearing loss, especially during cancer treatment.
